# A methodological approach to the study of archaeological cereal meals: a case study at Çatalhöyük East (Turkey)

**DOI:** 10.1007/s00334-017-0602-6

**Published:** 2017-03-16

**Authors:** Lara González Carretero, Michèle Wollstonecroft, Dorian Q. Fuller

**Affiliations:** University College London, Institute of Archaeology, 31-34 Gordon Square, London, WC1H 0PY UK

**Keywords:** Palaeoethnobotany, Near East, Food remains, Parenchyma, Neolithic

## Abstract

This paper presents an integrated methodology for the analysis of archaeological remains of cereal meals, based on scanning electronic microscopic analyses of microstructures of charred food fragments from Neolithic Çatalhöyük (Turkey). The remains of cereal foods as ‘bread-like’ or ‘porridge-like’ small charred lumps of various amalgamated plant materials are frequently recovered from Neolithic and later archaeological sites in southwest Asia and Europe. Cereal food remains have recently attracted interest because the identification of their plant contents, the forms of food that they represent and the methods used in their creation can provide unique information about ancient culinary traditions and routine food processing, preparation and cooking techniques. Here, we focus on three methodological aspects: (1) the analysis of their composition; (2) the analysis of their microstructure to determine preparation and cooking processes; (3) the comparison with experimental reference materials. Preliminary results are presented on the botanical composition and cooking processes represented by the charred cereal preparations found at Neolithic Çatalhöyük (Turkey), for example cereals processed into bread, dough and/or porridge.

## Introduction

Notwithstanding its significance for human health and fitness, food has received much attention from archaeologists, anthropologists and ethnographers because of its fundamental role in the diverse cultural, economic and political developments of human societies (Lévi-Strauss 1964; Goody 1982; Hastorf 1991, 1998; Dietler 1996; Sherratt 1999; Wright 2000; Dietler and Hyden 2001). Archaeobotany and zooarchaeology as disciplines for the study of how plant and animal resources were used by ancient communities have seen significant growth, including the development of methods for recognising how food preparation techniques may be represented by preserved remains, such as evidence for butchery marks, bone boiling or roasting (Speth 2012; Munro and Bar-Oz 2005; Koon et al. 2010; Russell and Martin 2012). Yet the forms in which prehistoric peoples actually consumed their plant food resources have only recently received scholarly attention (Stahl 1989; Hansson and Isaksson 1994; Samuel 1994, 1999, 2000; Hansson 2002; Valamoti 2002, 2011; Wollstonecroft 2007; Wollstonecroft et al. 2008; Valamoti et al. 2008; Heiss 2014; Kubiak-Martens et al. 2015).

The form and content of ancient meals is important because it can help us to better understand culinary traditions and associated routine activities such as processing and cooking, as well as providing information about their species selection and intensification practices (Stahl 1989; Wollstonecroft 2007) and the nutritional value of the food (Wandsnider 1997; Wollstonecroft et al. 2008, 2011). Archaeologists have typically inferred this type of information from indirect evidence such as associated artefacts and features, such as grinding stones, mortars and pestles, hearths, ovens and ceramics (Wright 1994, 2000; Moore 1995). Significantly however, the inferences of archaeobotanists and zooarchaeologists are based on the analysis of direct evidence, archaeological plant and animal remains. Of particular note are recent archaeobotanical studies of organic residues in food crusts from pottery that combine organic chemical analyses and observations by scanning electronic microscopy (SEM) (Raemaekers et al. 2013; Oudemans and Kubiak-Martens 2013; Kubiak-Martens et al. 2015).

Cereals were central to the economic transformations associated with the so-called ‘Neolithic revolution’ (Childe 1936). These were amongst the first plants to be domesticated worldwide (Fuller et al. 2014) and they are the staple food for a variety of ecological systems nowadays, with wheat in Europe and North America, maize in parts of Africa and South America, sorghum in India and parts of Africa and rice in East and Southeast Asia. It has been suggested that cereals, with their hard, dry and storable seeds were the key support to the rise of early states and the development of writing systems (Steensberg 1990). Nevertheless, the systematic study of the forms in which people consumed their cereal food, whether as whole grains, bread, porridge, bulgur, or otherwise, has been limited.

The most commonly recovered archaeological evidence of cereal products, however, is in the form of charred amorphous fragments collected with routine archaeobotanical flotation. These are isolated lumps of processed plant foods also called ‘bread-like’ or ‘porridge-like’ materials. This type of plant remain has often been disregarded by archaeobotanists due to the complex methodology and time consuming analyses needed for their successful identification. As a consequence, more easily recognisable materials, such as whole loaves of bread have frequently received the attention of archaeologists. In this sense, Währen (1989, 2002) was first among them to offer a typological classification of the different types of breads recovered from European archaeological sites from the Neolithic to medieval times, based on their morphology.

Hansson and Isaksson (1994) pioneered the tissue-based analysis of amorphous charred fragments of processed food remains from several archaeological sites in Sweden, Västergarden, Vrå, Harrsjöbacken and Folåsa. Their study was the first successful application of SEM to identify the biological composition of the charred remains, which they augmented with chemical analyses. Building on this, Hansson (2002) carried out detailed analyses of whole loaves of bread from other Swedish sites such as Birka, Helgö, Ljunga and Boberget. Subsequently, over the last two decades, numerous in-depth tissue-based analyses of prehistoric and ancient breads have been published. Deserving special mention are Samuel’s (1994, 1999, 2000) investigations of desiccated Egyptian bread from tombs in the New Kingdom (ca. 1550–1070 bc) which resulted in significant insights into ancient bread and beer making. More recent studies (Lannoy et al. 2002; Heiss et al. 2015), which focused on identifying the cereal components of Iron Age and Roman breads, demonstrate the usefulness of applying anatomical analysis to identify cereal tissue types; the results provide unique new information about the various ingredients used in bread preparation, as well as cereal processing techniques such as grinding, and various ways of preparing doughs and potential cooking practices. Kubiak-Martens et al. (2015) also applied SEM analysis to charred food crusts from pottery and isolated charred organic lumps recovered from late Neolithic sites in the northern Netherlands. Among the food ingredients, a range of plant-based cooked foods that included not only cereals but also fruits, nuts and tuber components were identified.

Another methodology was developed by Valamoti to investigate types of cereal foods that are not flour-based, such as bulgur wheat (Valamoti 2002; Valamoti et al. 2008). Ethnoarchaeological and experimental studies were combined on both cereals and pulses to investigate the form and composition of remains of cereal foods including observations of the intracellular features and changes in starch granule morphology.

Despite the recent increase in such studies, the analysis of archaeological remains of food is still very methodologically fragmented. A coherent methodology is needed to obtain a dependable assessment of taxa composition and possibly the identification of food processing methods from morphological changes to plant tissue (Antolin et al. 2016). It is thus the aim of this paper to present an integrated methodology for the analysis of archaeological remains of cereal foods. The methodology described here uses SEM observation to analyse the microstructures of amorphous charred food fragments from Neolithic Çatalhöyük, Turkey, which are compared with SEM observations of experimentally produced reference materials. We focus on three methodological aspects: (1) the analysis of their plant taxa composition; (2) the analysis of their microstructure to determine preparation and cooking processes; (3) the comparison with experimental reference materials.

## Neolithic Çatalhöyük plant subsistence

Çatalhöyük, a 9,000 year-old settlement mound (tell) site in central Anatolia, Turkey, provides an ideal site for the study of Neolithic cooking practices and style or method of cooking and selected sets of ingredients, as well as changes in those practices over time. This site had a long and continuous occupation (7100–6000 cal bc and beyond; Table 1), which spans the introduction of pottery and, in the later levels, the introduction of domesticated cattle and use of secondary animal products. A variety of domed ovens (*firin*) have been identified at Çatalhöyük dating from the late 8th millennium, and preceding the use of pottery as cooking vessels (Last et al. 2005). Çatalhöyük has also been extensively sampled for archaeobotanical remains, and produced rich and well preserved plant assemblages. Nevertheless, although food processing and cooking techniques at Neolithic Çatalhöyük were previously explored by Atalay and Hastorf (2006), the present study is the first methodological approach to the archaeobotanical analysis of charred food remains and their preparation at Çatalhöyük.

**Table 1:** Chronological system of levels at Çatalhöyük East and analysed charred fragments of processed plant food

Mellaart levels	Hodder levels	Phase	Chronology cal bc	Food samples
0, I, II	TP6	Late Neolithic	6400–6000	32
	South T—4040.J			–
	South S—4040.J			2
	South R—4040.I			2
	South Q—4040.H			4
V	South P—4040.H	Middle Neolithic		15
VIA	South O—4040.G	Middle Neolithic	6500–6400	12
VIB	South N—4040.G			13
VII	South M—4040.F	Early Neolithic	6700–6500	6
VIII	South L—4040.F			5
IX	South K	Early Neolithic	7100–6800	2
X	South J			–
XI	South I			–
XII	South H			3
Pre XII	South G1, G2, G3, G4	Pre-Pottery Neolithic		4

Çatalhöyük is located in south central Turkey on the Konya plain (37.6664°N, 32.8257°E). After its discovery in 1958, it was excavated by James Mellaart and his team from 1961 until 1965. Renewed excavations by *The Çatalhöyük Research Project* began in 1993 under the supervision of Ian Hodder. The site is divided into the East Neolithic Mound and the Chalcolithic West Mound, which represents the continuation of the occupation. As part of this project, our study focuses on material from the extensive occupation of the East Mound, dating from 7100 to 5950 cal bc (Bayliss et al. 2015). As a result of the last 20 years of excavation and research, Çatalhöyük is presently the most completely studied and detailed record of Neolithic households in the Near East. It is considered to represent a house-based egalitarian society in which every house in this Neolithic village had a similar role and function in routine activities such as daily food processing and food preparation (Hodder 2005, 2006, 2007, 2011, 2012, 2013).

Plant subsistence strategies are well understood there. Bogaard et al. (2013) highlight the importance of cereals and pulses as well as a core set of nuts and fruits as staple foods at Neolithic Çatalhöyük. The plant assemblage indicates consistent long-term use of six cereals: the hulled wheats *Triticum monococcum* (einkorn), *T. dicoccum* (emmer) and ‘new’ type glume wheat, described by Jones et al. (2000) and by Kohler-Schneider (2001, 2003), *Hordeum vulgare* var. *nudum* (two- and six-row naked barley) and *T. aestivum* (bread wheat). Three pulses were also used, *Pisum sativum* (pea), *Lens culinaris* (lentil) and *Vicia ervilia* (bitter vetch). Also used were oily-seeded *Descurainia sophia* (flixweed), three nuts, *Prunus dulcis* (almond), *Quercus* sp. (acorn), *Pistacia vera* (pistachio) and two fruits, *Celtis* sp. (hackberry) and *Ficus carica* (fig). This core set of staple foods confirms that these early crops were used in combination at the household level, reflecting cultivation of annual seed crops alongside gathering of wild resources. While some of these assemblages can be inferred to have come from the use of animal dung as fuel, others are more directly related to the waste from human activities such as crop processing waste (Bogaard et al. 2013; Filipović 2014). Fairbairn et al. (2005) also demonstrated that residues raked out of fireplaces are the most concentrated deposits of charred plant material produced through routine practices; these primary deposits were subsequently re-used and deposited as secondary contexts in middens and as tertiary contexts in building fills and construction material.

## Materials and methods

**Fig. 1:**
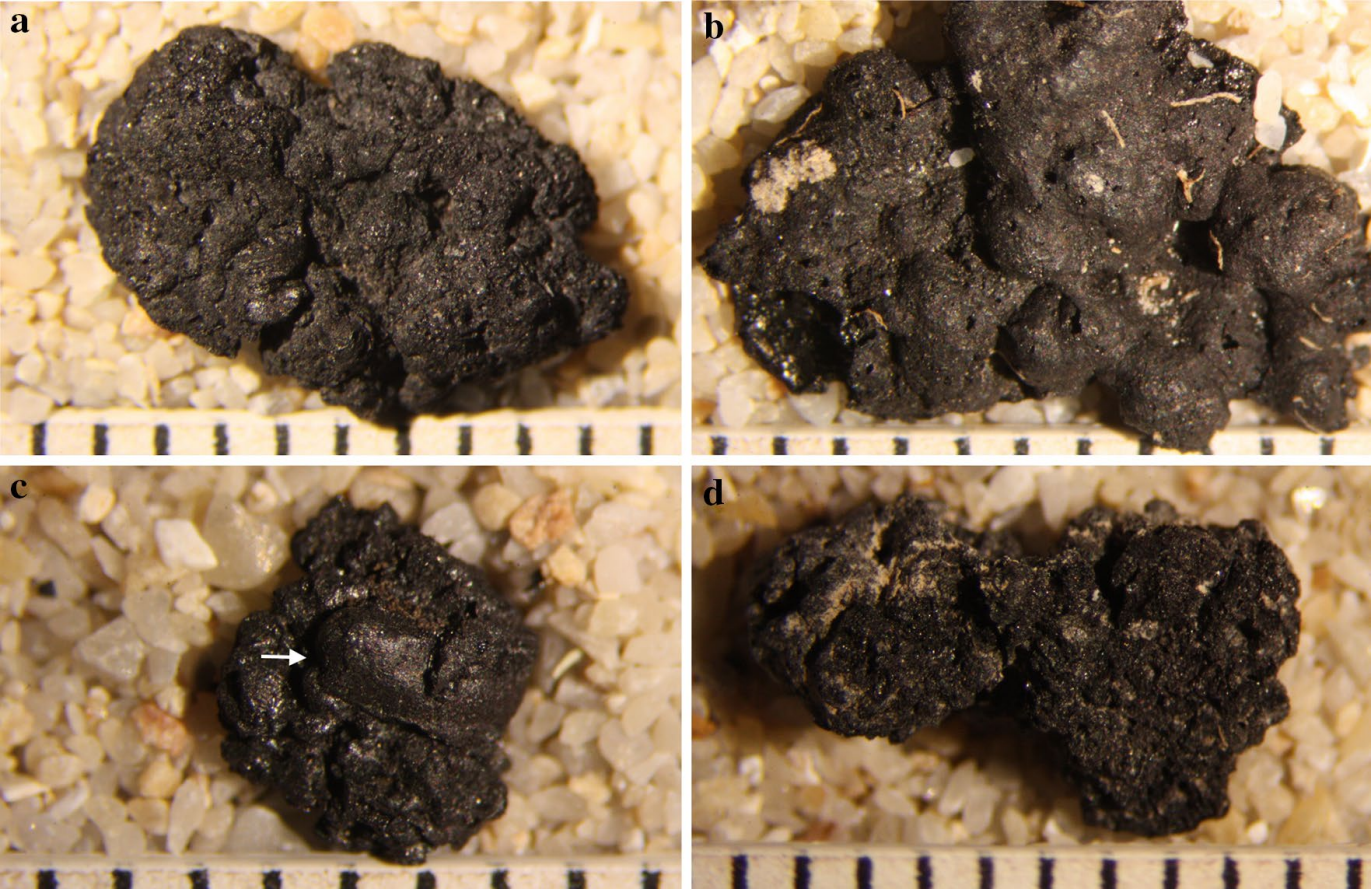
Fragments of processed plant food from Çatalhöyük East: **a** Fl.10661; **b** Fl.11860; **c** Fl.10721; **d** Fl.9875

Among the macrobotanical remains present at the site, amorphous charred fragments of cereal products were among the ones most frequently recovered during sorting of flotation samples (Bogaard et al. 2013), but were not previously subjected to any systematic examination (Fig. 1).

More than 200 charred food samples, which span the chronological sequence, were collected from sorted flotation samples during the 2013–2015 excavation seasons at Çatalhöyük (Table 1; Filipović et al. 2014; Bogaard et al. 2015). The present paper summarises results of the study of 100 of the samples and illustrates a systematic methodology and terminology for investigating them.

### Field sampling

Due the lack of previous methodology applied to the recovery of archaeological food remain samples, a new systematic procedure for their collection on site was developed for this study. For the initial recovery of macrobotanical remains, we followed sampling and processing methods developed during the 1990s excavations, including machine flotation and wet sieving (Hastorf 2005). Random subsamples of the 1–4 mm fraction of the flot were extracted with a riffle-box for sorting of an initial subsample of ca. 10 ml to provide a first quantitative assessment of seed and chaff content, while the >4 mm fraction was scanned for non-wood charred remains such as nuts, tubers and food fragments.

Flot samples which contained amorphous charred cereal remains during assessment were selected for further analysis. 200 flots containing these remains, from contexts that span the major phases of Çatalhöyük, were selected from the North, South and TP (Team Poznán) areas of the site during 2013, 2014 and 2015 excavation seasons. Initial scanning on site suggested that these samples were mainly representative of in situ food processing contexts such as ovens, hearths, fire spots and storage deposits as well as representing aspects of ‘Neolithic recipes’ before, during and after cooking. These samples were then sorted in their entirety of >1 mm seed remains and smaller fractions were subsampled and scanned.

### Microscopy

Several techniques have proved successful in previous research for the analyses of archaeological charred food remains or cereal products, from morphological to micro-anatomical tissue analysis and the application of chemical methods (Währen 1989, 2002; Hansson and Isaksson 1994; Kubiak-Martens et al. 2015). We chose to apply an integrated methodological approach which combined (1) microscopy: the study of microstructures and plant cell tissues under a low-powered binocular microscope; (2) more detailed characterisation with a scanning electron microscope, including the study of the food matrix through semi-quantitative recording of voids and particles, and anatomical description of any included recognisable plant tissues; and (3) the experimental preparation of cereal foods to use as reference materials for comparison with the archaeological charred food remains.

Initial observation of the samples under a low-powered microscope was made using a Leica EZ4 binocular microscope at magnifications of ×8 to ×40; images were created using a Leica S6D microscope and a Leica EZ3 camera. From these, food fragments that showed visible plant inclusions such as plant tissues were selected for further study under SEM. For SEM observation, the samples were cleaned from soil sediments with a brush, sputter coated with ca. 20 µm of gold, and examined using a Hitachi S-3400N scanning electron microscope. During the microscopic analyses, two main aspects were investigated: the identification of specific types of plant tissue in order to clarify the ingredients used for the preparation of these foods and also the exploration of their microstructures, which are a result of the processing and cooking techniques used for their preparation.

The first aspect of investigation is based on identification characters developed by Dickson (1987), Colledge (1988), Holden (1990) and Heiss et al. (2015) and botanical reference materials in the UCL Institute of Archaeology plant reference collection. Therefore the following plant tissues were considered, as previously tested by Heiss (2010, 2012) and Heiss et al. (2015): tissue layers present in the cereal grains (pericarp and seed coat), chaff (epidermis of paleas and lemmas), other parenchyma tissues (pulses and tubers), vascular tissues (tubers) and starch granules. These last, which are not easily preserved in charred material, can provide important information about processes used in food preparation (Samuel 1994; Valamoti 2002; Valamoti et al. 2008).

We are aware of no previous example of a cohesive and systematic methodology for the study of microstructures of amorphous charred food fragments, apart from the previously mentioned work by Hansson and Isaksson (1994) and brief mention of possible cooking processes in relation to their shape and consistency (Lannoy et al. 2002). Thus one of the main aims of this study was to create a specifically designed methodology for the study of microstructures of archaeological food remains. Two features in particular were chosen for further exploration: (1) cooking processes which could have created fewer or more pores (voids) in the food matrix and affected the quantity, shapes and types of voids; (2) various pre-cooking processes, such as different types of grinding techniques or various water contents and the cultural choices of ingredients which directly determined the types of particles present in the food matrix, and how they were preserved in the charred remains.

### Typological data sets for the analysis of microstructures of archaeological cereal products

For the creation of reliable and accurate typological data sets for the analysis of the internal structures of archaeological charred cereal products, typological work on the description of different archaeological materials such as soil, sediments and pottery was explored. Based on comparison of pottery with cereal products such as bread, as the process in which ‘a shapeless mass is first prepared, then given form, before being cooked in a kiln’ (Lévi-Strauss 1988), we chose to build on the principles developed to study pottery fabrics in relation to porosity, hardness and inclusions, as developed for example by Washburn (1921), Grimshaw (1971), Rice (1998) and Mathew et al. (1991). In particular, voids are characterised to assess the nature of porosity, and included particles such as non-plastic inclusions in a pottery matrix can be characterised in terms of size, distribution and angularity of inclusions. In the case of food, these inclusions often contain anatomically recognisable fragments of plant cell tissues, but may also represent transformed amorphous starch.

**Fig. 2:**
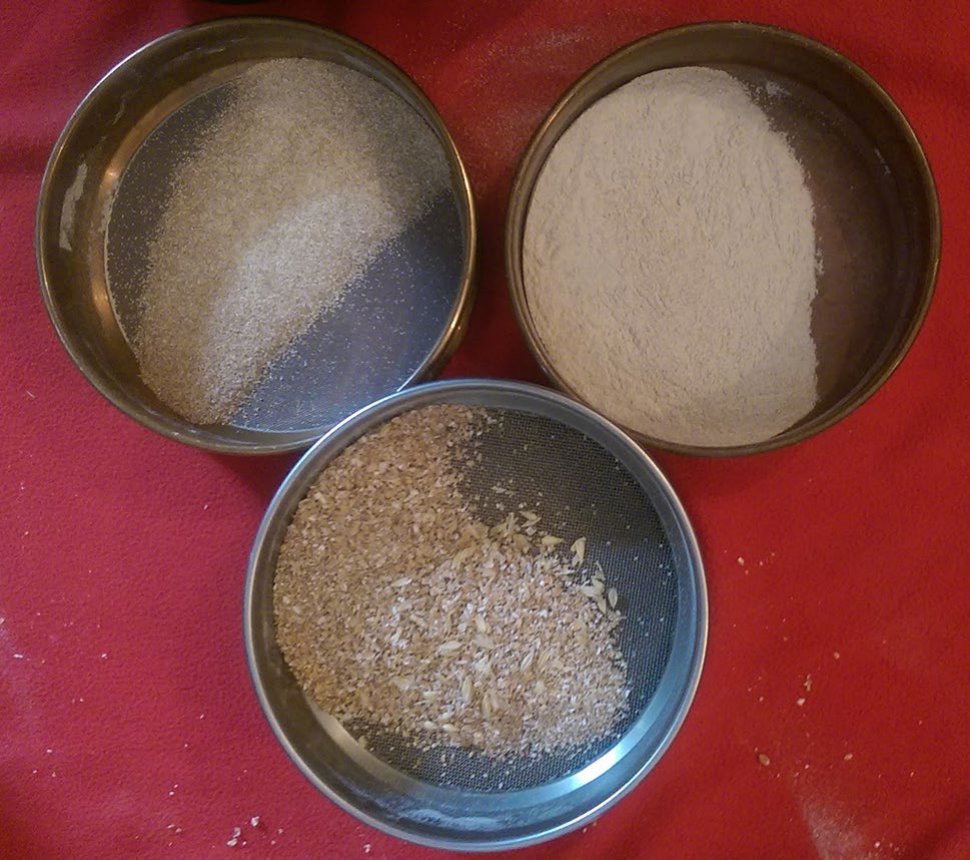
Experimental flour types: fine (<0.5 mm), coarse grain or fine bulgur (>0.5 mm) and coarse bulgur (>1 mm) mixed with cracked grain and flakes

**Fig. 3:**
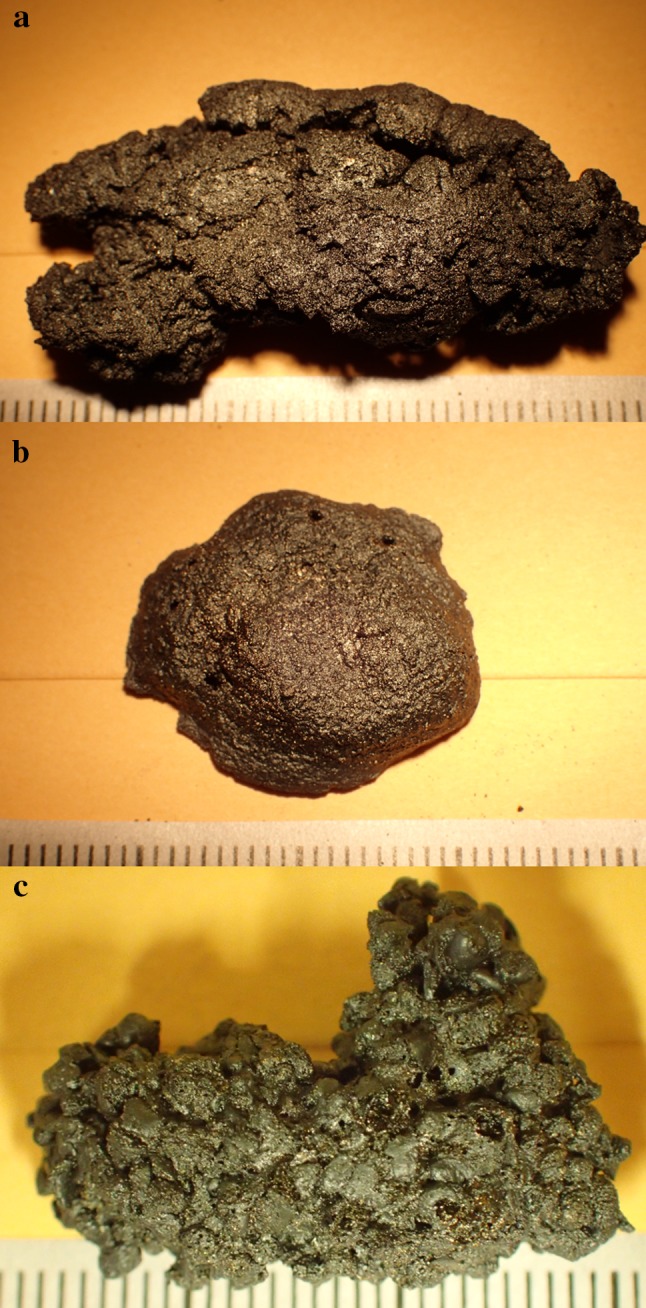
Fragments of experimentally prepared cereal foods, charred at 300 °C for 3 h: **a** no water treated wheat flat bread; **b** no water treated wheat dough, **c** no water treated wheat porridge

**Fig. 4:**
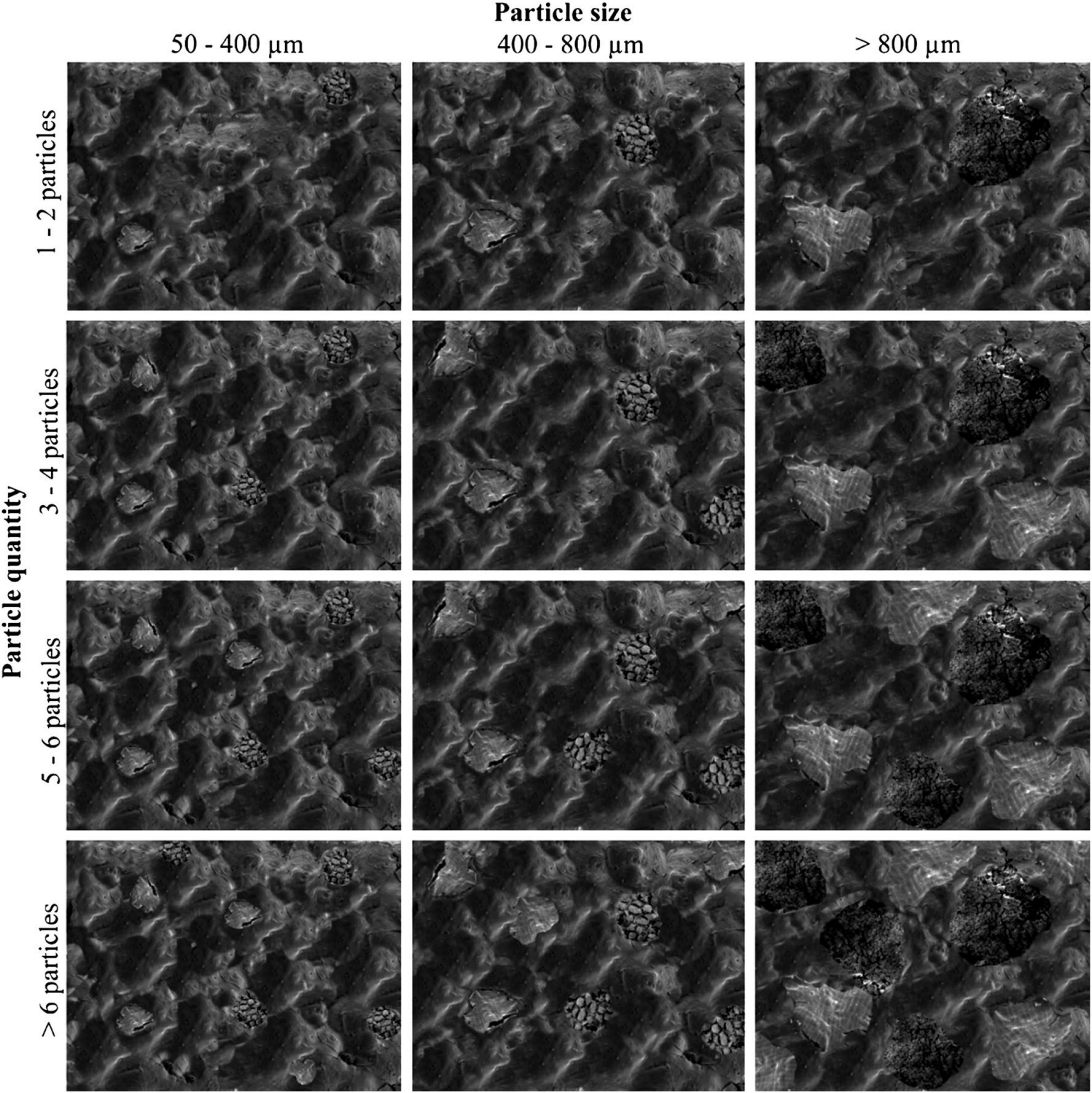
Attribute set A: particles. Estimation chart based on size and quantity of visible plant particles in the matrix

Three attribute sets are used to describe the internal structure of the archaeological charred cereal food remains:

- *Attribute set A* focuses on the quantification and measurement of visible plant inclusions or particles in the food matrix, such as cereal tissues (Fig. 2).
- *Attribute set B* focuses on the estimation, quantification and measurement of voids from air or gas bubbles in the food matrix (Fig. 3).
- *Attribute set C* focuses on the typological classification of the previously quantified voids, in terms of void types and shapes (Fig. 4).

Following the different attribute categories described above, 100 food remains fragments were analysed by SEM for specific observation of the matrix microstructures. During this, up to six images of the matrix of a food fragment were captured, at 16–20 mm working distance depending on fragment size and at ×50 magnification, in order to cover the whole surface. Then the particles (Attribute A) and voids (Attributes B and C) were estimated, quantified, measured and categorised following a simple process.

First, visible particles or inclusions in the ×50 magnification image were counted and the average was calculated when there was more than one image. Then each particle length was measured using the SEM Hitachi measurement tool and the average length of the particles was calculated.

Second, using the estimation chart, gas bubbles or voids visible on the ×50 magnification images were quantified and measured. Voids were estimated, depending on their size and the percentage of matrix surface that they covered. Following the categories created on the charts, a mean size and an estimated percentage of voids in the food matrix were calculated. To minimise error, the percentage of voids was estimated again using Gwyddion, a free access modular program for SPM (scanning probe microscopy) data visualization and image analysis (by David Nečas and Petr Klapetek, Department of Nanometrology, Czech Metrology Institute). This allowed confirmation of the accuracy of estimates made by eye.

Third, the matrix was classified according to the shapes of the voids, based on the most frequent void shape encountered on the ×50 magnification image.

### The preparation of experimental reference materials

Experimental cooking and charring experiments are a major part of the methodology for this research study. Experimentally prepared and charred foods, which were created under controlled conditions, will serve as a reference collection for identifying the contents of the carbonised archaeological food fragments under study. Due to the previous identification of Çatalhöyük charred foods as “bread” (Bogaard et al. 2013) and also because of their frequent visible cereal inclusions in the matrix, cereal foods were chosen for this first set of experiments.

Different cereal grain materials were selected. Two types of cereals, wheat and barley, which are present in the archaeobotanical record at Çatalhöyük East (Bogaard et al. 2013) were chosen for this first set of experiments. *Triticum aestivum* was harvested in Dorset (UK) and cleaned prior to processing, while *Hordeum vulgare* was purchased already cleaned from fields in Germany through the fairtrade company Biolandhof Knauf. Both types of grain received three different treatments followed by different types of processing into three cereal products:

- 200 ml not water treated wheat or barley grain → ground into flour → dough, bread and porridge
- 200 ml soaked (12 h) wheat or barley grain → air-dried → ground into flour → dough, bread and porridge
- 200 ml boiled (15 min) wheat or barley grain → air-dried → ground into flour → dough, bread and porridge.

#### Grinding

**Fig. 5:**
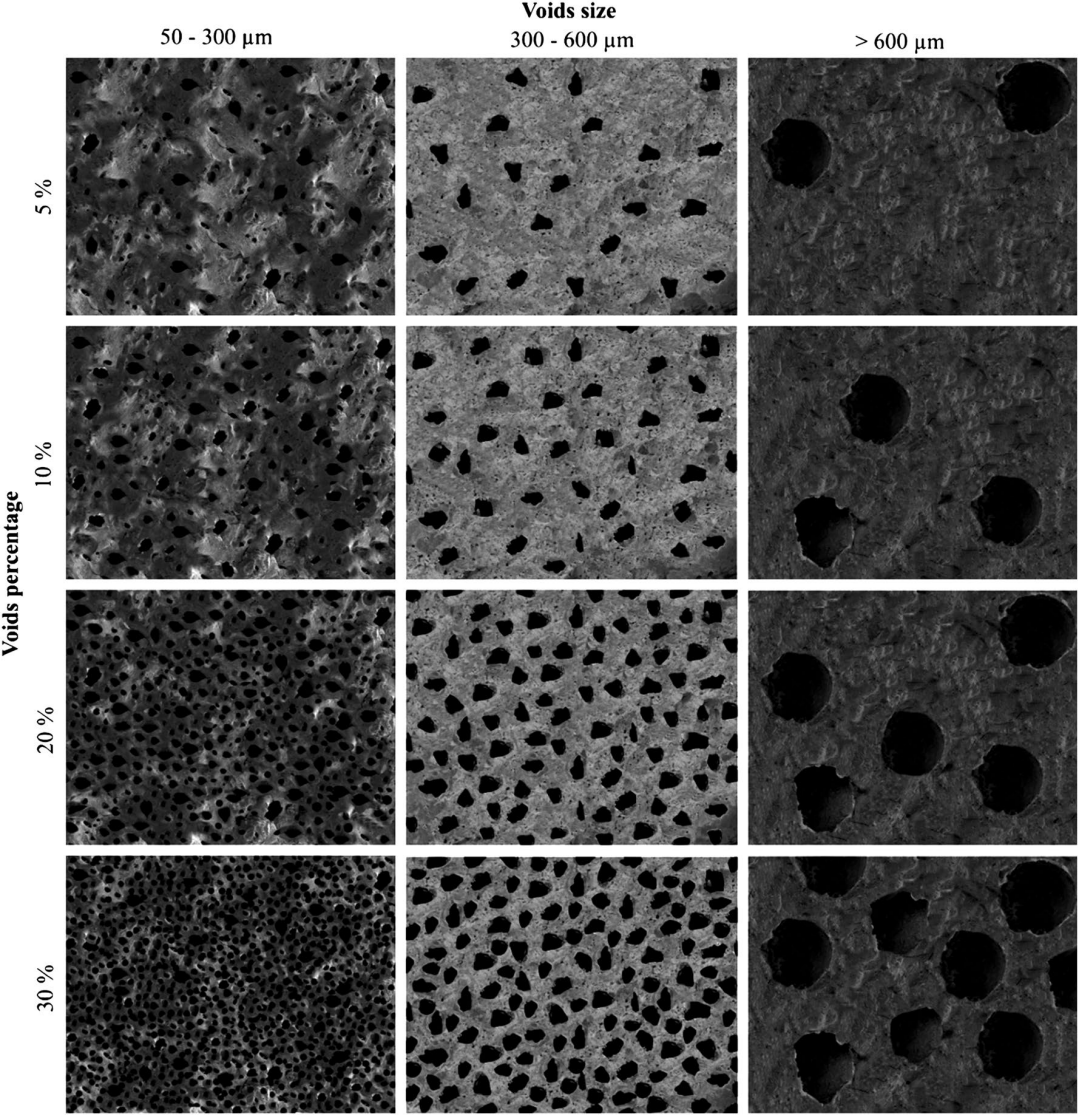
Attribute set B: voids. Estimation chart based on size and percentage of voids (air bubbles) in the matrix

Grinding was done until a majority of fine flour was produced (45 min to 1.5 h), using an andesite hand stone and quern measuring 40 × 18 × 11 cm. To grind, a small handful of grain was placed on the middle of the quern and short strokes with the hand stone broke down the grain and easily ground it into flour. Soaked, boiled and dry wheat and barley grain were all ground to fine flour using this technique. After grinding, the flour was sieved using two different geological sieves, 1.0 and 0.5 mm, the mesh sizes of which resemble ethnographical examples. Three different types of flour resulted from the sieving: fine (<0.5 mm), coarse grain or fine bulgur (>0.5 mm) and coarse bulgur (>1 mm) mixed with cracked grain and flakes (Fig. 5).

#### Preparation

These three flours were then prepared into dough, flat bread and porridge or gruel following traditional methods previously observed in modern Turkish villages (Hillman 1973, 1981, 1984; Yakar 2000). For the preparation of the dough, 15 ml of water was added to 25 ml of a mixture of fine flour (<0.5 mm) and coarse grain (>0.5 mm). The different dough types were then kneaded for approximately 1 min. For the preparation of the flat breads, 80 ml of fine flour was mixed with 30 ml of water and kneaded until the consistence of bread dough was reached. The dough was then put on a baking tray and baked at 180 °C for 30 min using an electric oven. For the preparation of porridge, 100 ml of the coarser meal produced (>1.0 mm) during grinding was added to 100 ml of boiling water, then the mixture was boiled for 3 min until the consistence of a paste or gruel was reached and the water evaporated.

#### Charring

**Fig. 6:**
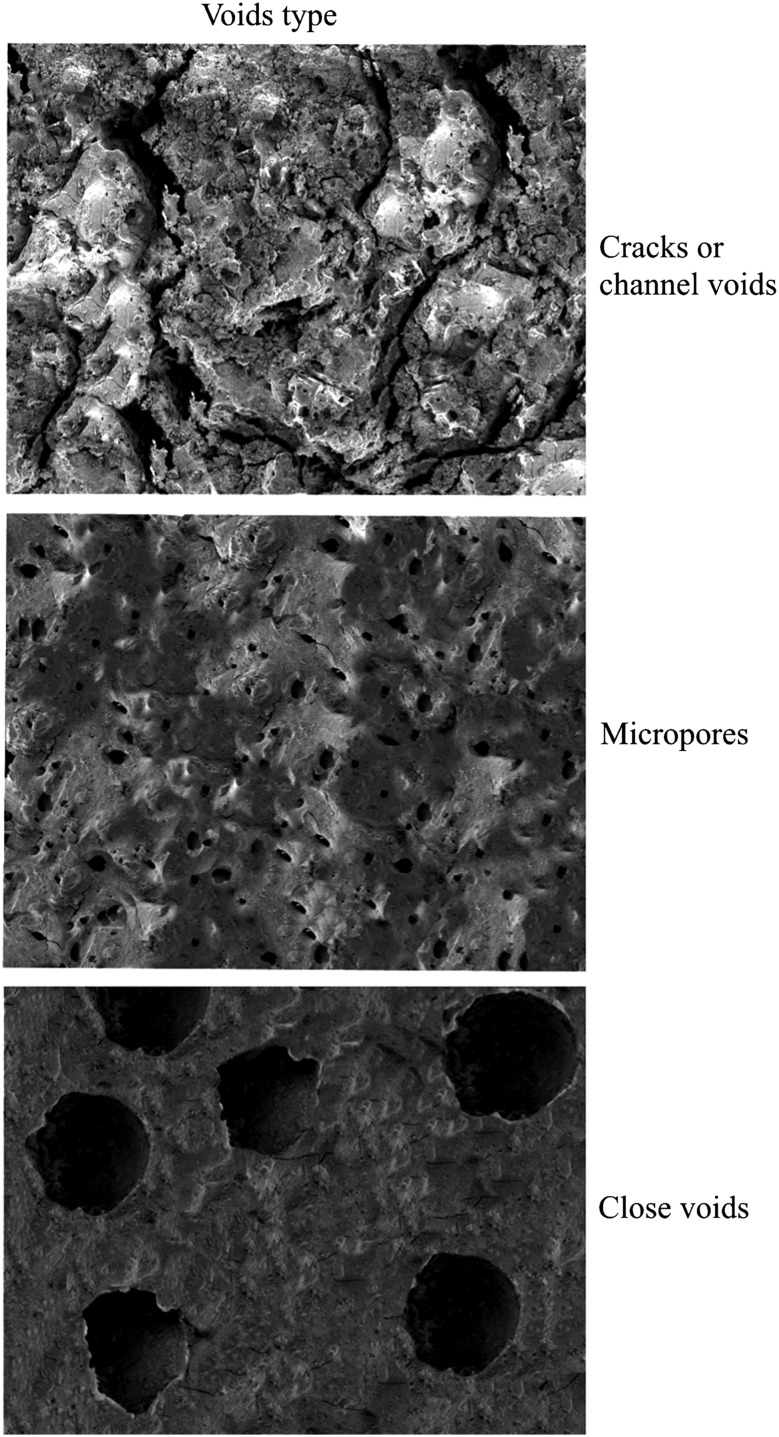
Attribute set C: type of voids. Estimation chart based on shape of voids (air bubbles) in the matrix

The charring experiments on the prepared cereal foods were then conducted using a muffle furnace at the Institute of Archaeology at University College London. Three similar sized pieces (30 × 15 × 10 cm) of each cereal preparation were charred at 300 °C for 1, 2 and 3 h in order to recreate a possible intentional burning and to understand the behaviour of the matrix, starches and tissues when charred for different times in the archaeological food remains (Fig. 6).

These experimentally created charred food specimens were then studied under low powered binocular microscope and SEM in order to compare the starch granules, plant tissue remains and matrix internal microstructure with the archaeological food remains from Neolithic Çatalhöyük. These charred specimens are the first set of materials which will form part of an extensive reference collection of charred food materials to help the future investigation into archaeological food remains. This will not only include a variety of cereal foods but also other food preparations such as legume flours and others.

## Results

### Components

Of the 200 archaeobotanical flotation samples selected from Neolithic Çatalhöyük, 172 contained charred food fragments that have provided information about their plant food components. The amount of archaeological food per sample varied between 0.1 and 20 ml and the sizes of these fragments were from 0.1 to 2.4 cm. These food fragments were all seen to have a starchy microstructure and irregular porous matrix, which together indicate well-processed and cooked plant components, with cereals as the main ingredient. Seen under the binocular microscope, plant remains, mainly cereal components and tissues, appeared as small shiny areas on the charred food materials. However, identification of specific tissue types and taxa was only possible by SEM.

For this study, 100 charred food fragments have been fully analysed under SEM and categorised according to the typological datasets explained above. Almost all samples chosen are from primary deposits, mainly burnt in situ contexts, and they cover the full Neolithic sequence at Çatalhöyük East, from the earliest level on site, Hodder Pre-pottery South G (Mellaart Level Pre XII), to the TP S Hodder Level (Mellaart Level 0) which is considered the latest Neolithic level found to date. From the 100 food fragments, 93 have provided information about their plant food components, with 91% of the remains comprised of cereals (wheat and barley).

**Fig. 7:**
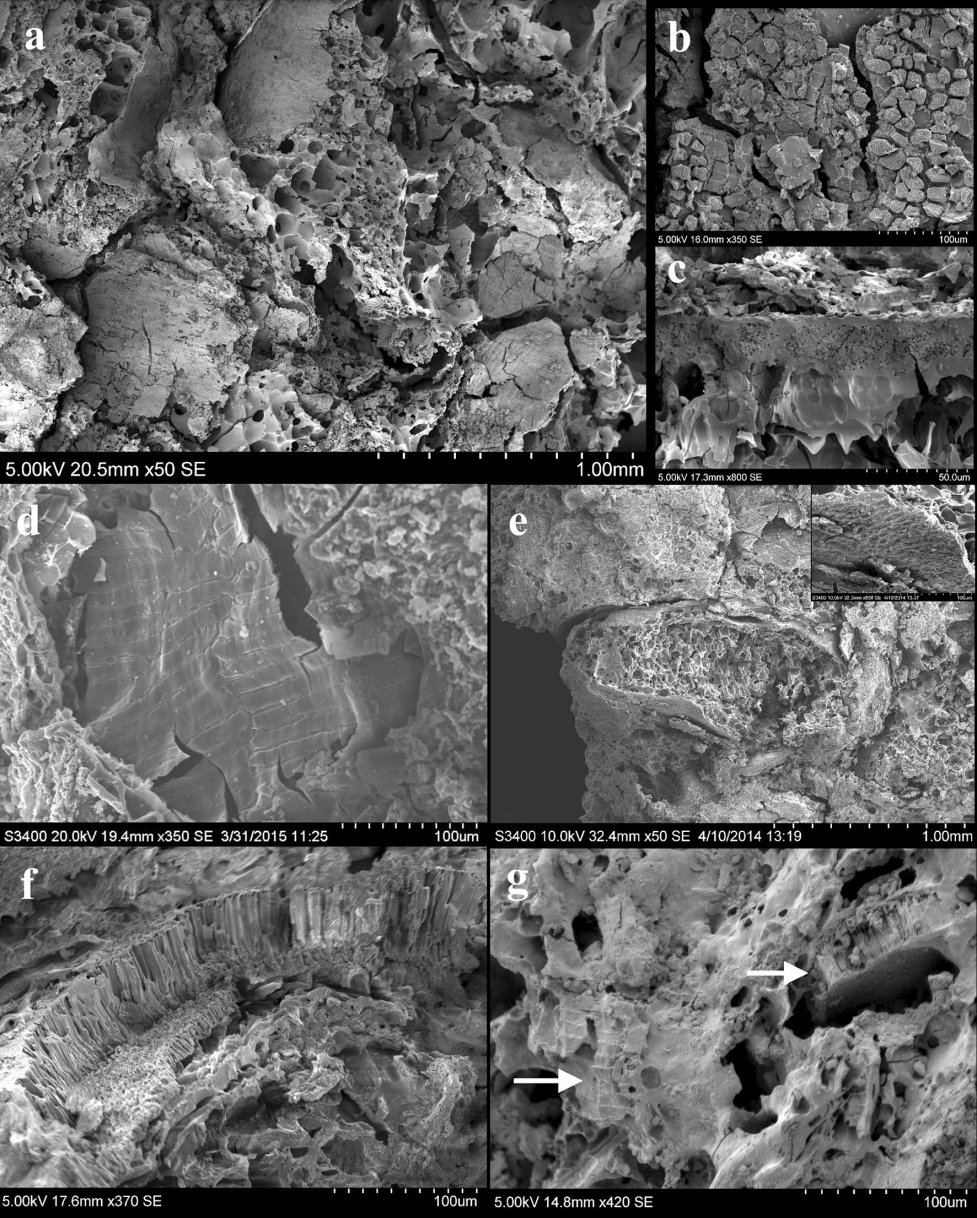
SEM micrographs showing plant components in food fragments: **a** Fl.9822, *Hordeum* grain and bran fragments embedded in food matrix; **b** Fl.7860, remains of aleurone layer from food fragment; **c** Fl.10742, unicellular aleurone layer from food fragment; **d** Fl.6939, fragment of cf. *Triticum* bran transverse cells; **e** Fl. 3099, *Descurainia sophia* seed embedded in food matrix and detail of seed coat; **f** Fl.11137, remains of visible pulse palisade cells layer; **g** Fl.11240, remains of pulse palisade cells layer and cereal bran, marked by *arrows*, mixed together in food matrix

There is a general consistency in the plant food components of the 100 food fragments. To assess the probability of these being remains of cereal foods as suggested by previous observation (Bogaard et al. 2013), we chose to separate visible plant tissues into different categories. These categories are based on a clear distinction between cereal and non-cereal components. The majority of identified tissues are internal tissues of the cereal grain. Among these, pericarp tissues such as bran layers (transversal and longitudinal cells) and endosperm cell structures such as aleurone layers and endosperm starch containing cells were observed (Fig. 7).

Only 8% of the components observed under SEM were non-cereal components, some of which were recognised as fragments of pulses, based on the identification of the distinctive *Vicia ervilia* and *Lens culinaris* testa patterning in samples Fl.3099, Fl.10770, Fl.10798, Fl.11137, Fl.11235 and Fl.11240 (Fig. 7f, g; Butler 1990). Other non-cereal components have been identified as “wild” food components such as *Descurainia sophia* which is present at Neolithic Çatalhöyük in large quantities in storage and cooking contexts, from sample Fl.3099 recognised by the distinctive shape and seed coat (Fig. 7e). Also, the material from finely ground sedge tubers was given the preliminary identification to the species *Bolboschoenus glaucus* in samples Fl.7928, Fl.8641 and Fl.10664, as suggested by the shape and organisation of parenchyma cells and vascular bundles (Wollstonecroft et al. 2008, 2011).

In some cases, an identification of cereal components to genus level (wheat and barley) has also been possible, due to their cell and tissue shapes, sizes and patterning. Among the recorded components, visible broken grains, bran fragments and patches of aleurone tissue were very abundant:

- *Fragments of cereal grains* broken cereal grains were found in 22 of the 100 analysed food fragments. The identification of a broken grain of *H. vulgare* var. *nudum* was possible in Fl.9822 due to its distinctive shape, rippling surface and a fragmented double-layered aleurone (Fig. 7a). Also in Fl.10967 and Fl.11177 two fragments of *H. vulgare* grains were identified from the specific disposition of bran transverse cells measuring ~30–70 by 10–20 µm on the surface (Dickson 1987). Wheat grains have also been identified; one almost complete (cf. *Triticum*) in Fl.10661 and a fragmented specimen exhibiting the grain cross-section with single-layered aleurone and endosperm cells in Fl.3099.
- *Bran* fragments of bran, which included longitudinal and transverse cells, were by far the most commonly found particles among the food remains. They occurred in 85 of the 100 analysed samples, from 50 to 1,200 µm in size. The distinction between cf. *Triticum* and *Hordeum* sp. transverse cells was made following criteria established by Dickson (1987), Holden (1990) and Colledge (1988). Good examples of these components were found in a 1.5 mm area of long and narrow cf. *Triticum* transverse bran cells (70–280 × 10–18 µm) from Fl.3666 (Fig. 7d) and shorter transversal cells (30–70 × 10–20 µm), distinctive of *Hordeum* species from Fl.10553.
- *Aleurone tissue* fragments of both single-layered aleurone tissue fragments as in wheats, oats, millets and rye and multi-layered ones (only in barley) were found in 61 of the 100 analysed food fragments. Fl.3099, Fl.6939, Fl.9924, Fl.10570 and Fl.10661 contained single-layered aleurone tissues that are distinctive for wheat (Fig. 7b, c); in Fl.9822 and Fl.9016, a barley double-layered aleurone is clearly visible.

In addition, a small quantity of cereal chaff was identified among the food fragments. Partial remains of glume epidermis could be seen in Fl.3666 and Fl.9016 with no further species identification; a complete barley rachis was visible in Fl.9822. Its identification was possible through the observation of the typical *Hordeum* glume epidermis “twin” arrangement of two short cells with a larger crescent shaped cell encircling a smaller circular one (Winton and Winton 1932).

### Morphology and microstructure

Once we applied the typological datasets created for the present study of archaeological food remains, we were able to distinguish four different types of internal structures in the matrices of the materials recovered from Neolithic Çatalhöyük (Table 2):

**Table 2:** Matrix correlation between experimental and archaeological food samples

Experimental	Archaeological	Attribute A	Attribute B	Attribute C
Dough	Matrix 1	0–2; 50–300 µm	>30%; 500–800 µm	Close voids
Flat bread	Matrix 3	1–3; 50–300 µm	5–10%; 100–200 µm	Micropores
Porridge	Matrix 4	>6; 500–1,300 µm	10–20%; 200–500 µm	Cracks, channel voids

**Fig. 8:**
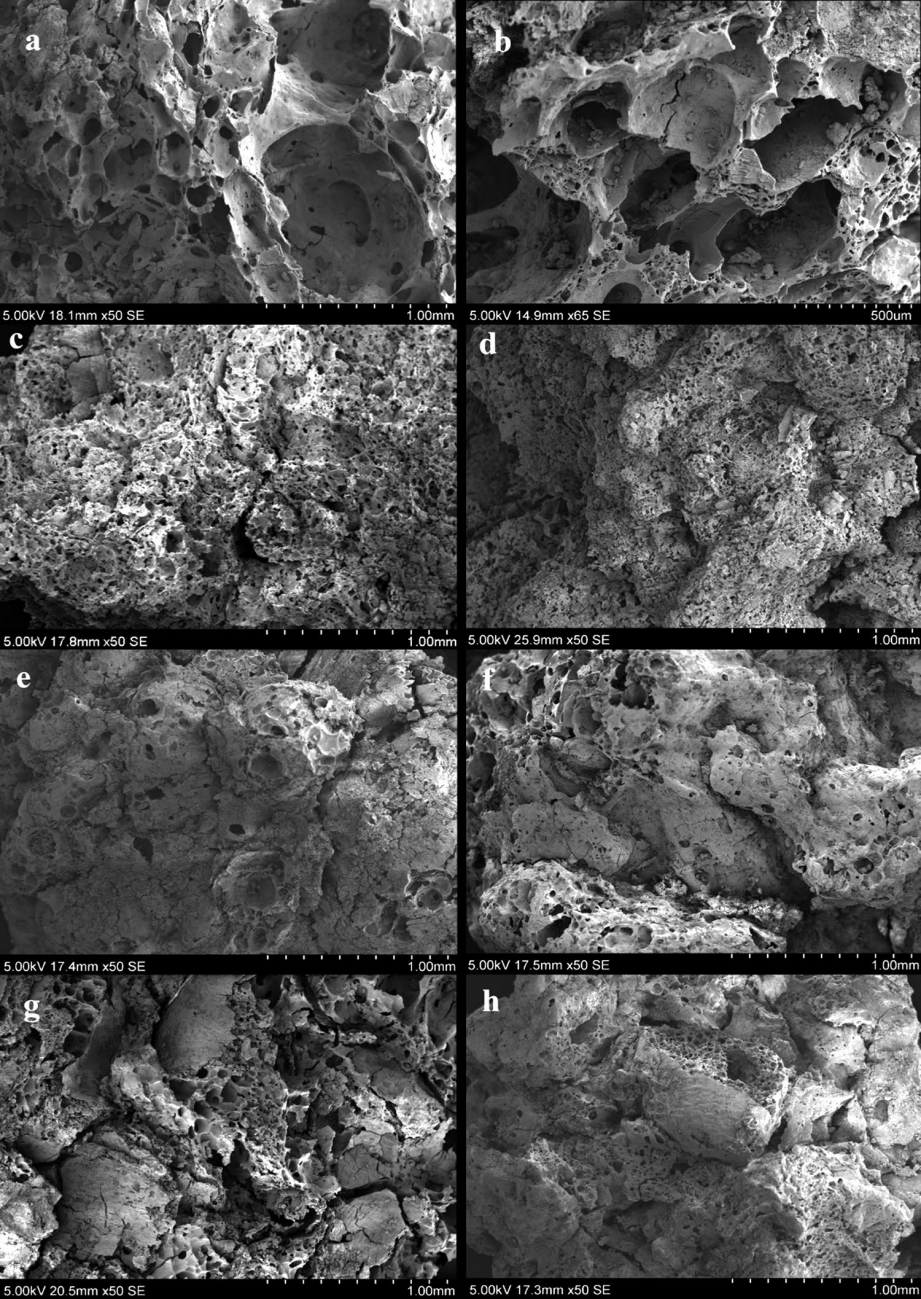
SEM micrographs showing different food matrices identified from processed foods at Çatalhöyük East: **a, b** Matrix type 1; **c, d** Matrix type 2; **e, f** Matrix type 3; **g, h** Matrix type 4

- *Matrix Type 1* Very few and small visible particles (0–2; 50–300 µm) and large close voids (see typological attribute C) from 500 to 800 µm which cover a high percentage of the microstructure surface (>30%) (Fig. 8a, b).
- *Matrix Type 2* A few small visible particles (1–3; 50–300 µm) in which the air bubbles or voids are micropores (100–200 µm) and cover more than 30% of the surface (Fig. 8c, d).
- *Matrix Type 3* A few small visible particles (1–3; 50–300 µm) and a low percentage of micropores (100–200 µm), covering between 5–10% of the surface (Fig. 8e, f).
- *Matrix Type 4* Lumpy matrix with a very large number of large visible particles (> 6; 500–1,300 µm) and a medium percentage of large cracks and channel voids (10–20%) (Fig. 8g, h).

The first three types of matrices are characterised by a few (0–3) small visible particles (50–300 µm) and have been observed in the food remains from the South and North areas at Çatalhöyük East from Levels South G to South S (7000–6400 cal bc). On the other hand, the fourth type of matrix which contains larger (500–1,300 µm) and numerous (>6) visible plant particles including grain fragments, large areas of cereal bran, etc., has so far only been found among the food fragments recovered from the latest levels at the site, in the TP areas (6400–6000 cal bc).

### Comparison with experimental materials

The first set of experiments in the present study provides insights into the Neolithic food processing and cooking techniques at Çatalhöyük East.

Firstly, the pre-processing water treatments of the grain by soaking and boiling it affected the subsequent grinding process. Grain that was soaked or boiled became soft and showed higher resistance to grinding, making it more difficult to produce fine flour. Conversely, dry grain was easily ground into fine flour. Additionally, during the grinding experiments, *H. vulgare* proved to be more productive, resulting in 20% more fine flour than modern *T. aestivum*. Another observed advantage of *H. vulgare* over *T. aestivum* is that, when under the same environmental conditions, soaked and boiled *Hordeum* dried out in less than 2 h, while soaked and boiled *Triticum* never dried completely.

Secondly, during the preparation of the three different types of cereal foods, dough, bread and porridge, the flour produced from dry grain was more suitable for bread making, but flour produced from wet grain appeared compacted and was more difficult to prepare into soft and elastic dough. Bread wheat proved to be more suitable for bread and dough making than *H. vulgare* since due to its high gluten content *T. aestivum* provides the necessary viscosity (thickness and texture) and elasticity to the mix. For the preparation of porridge however, boiled and soaked grain was shown to be better, taking less time to soften and producing a smoother paste. No clear differences in behaviour between wheat and barley were found during the preparation of porridge.

Lastly, during charring, although no real differences in behaviour were found between cereal taxa, the prepared cereal products responded differently to charring. While doughs completely carbonised during the first hour in the furnace, breads and porridges needed at least 2 h to char. These disparate behaviours are probably due to differences in water content of the foods. Also, the charring of doughs resulted in the formation of a crust on the outside and a hollow matrix inside; conversely, charring the breads resulted in almost no differentiation between crust and crumb.

**Fig. 9:**
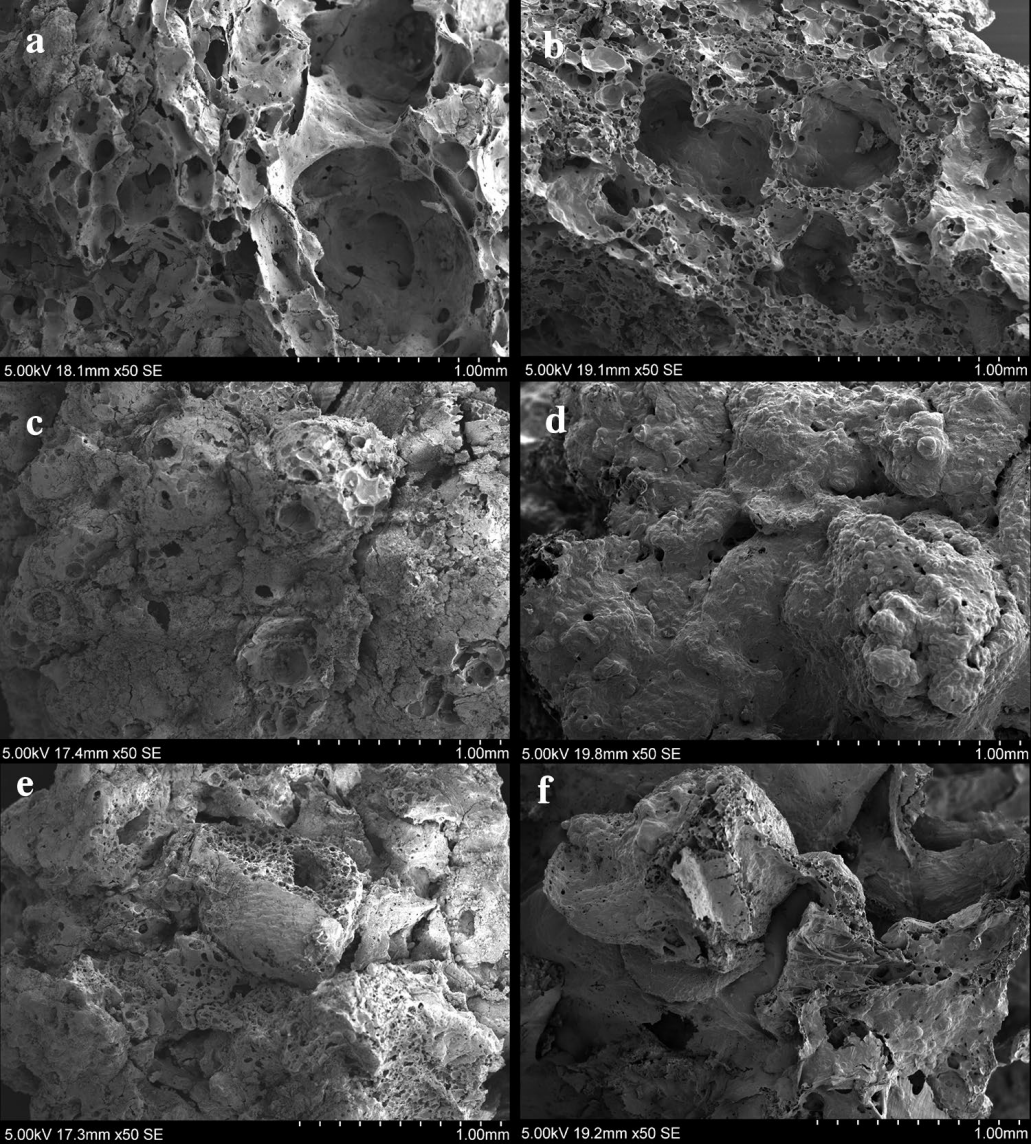
SEM micrographs showing correlation between matrices from archaeological (*left*) and experimentally prepared food samples (*right*) : **a, b** correlation between Matrix type 1 and experimental doughs; **c, d** correlation between Matrix type 3 and experimental flat breads; **e, f** correlation between Matrix type 4 and experimental porridges

The most significant results are thus from the comparison of the experimental charred cereal foods with the archaeological remains. Using the binocular microscope and subsequently the SEM, we observed striking similarities between the internal structures of the experimental foods and archaeological specimens. A close analysis following attribute B (void number and size) and attribute C (type of voids), revealed that experimental dough, bread and porridge had three different types of internal structure, which were almost identical to Matrix 1, Matrix 3 and Matrix 4 seen in the archaeological food fragments:

- *Doughs* had a hollow matrix, with large close voids (see Attribute C), 500–800 µm, which covered more than 30% of their surface. This structure corresponded to Matrix 1 (Fig. 9a, b).
- *Flat breads* had fewer micropores (see Attribute C), 50–250 µm and which covered between 5–10% of their matrix. This structure corresponded to Matrix 3 (Fig. 9c, d).
- *Porridges* had a distinctive internal structure with channel voids and cracks (see Attribute C), which varied between 200 and 500 µm in size and covered between 10 and 20% of the matrix. This corresponded to Matrix 4 (Fig. 9e, f).

Moreover, the same range of visible plant food particles, such as bran or grain fragments, was also found in the experimental specimens, with a marked correspondence in quantity and size among matrix types. For instance, following Attribute A (particle quantity and size), experimental doughs and flat breads had a small number of visible particles (0–3) which varied in size 50–300 µm; while porridges had a large number of visible food particles (5–10) with sizes between 500–1,500 µm.

## Discussion

### Components

Among the ingredients identified in the food remains, there is a consistency in the presence of domestic staple foods such as cereals and pulses in 93% of the analysed fragments. Among these, four pulses (*Lens culinaris, Cicer arietinum, Pisum sativum* and *Vicia sativa*), *H. vulgare* and *Triticum* spp. were the main ingredients of Çatalhöyük food remains. However, among the 100 analysed food fragments from Neolithic Çatalhöyük we also found two wild plant ingredients, the extensively used *Descurainia sophia* (Fairbairn et al. 2007; Bogaard et al. 2013) which has been identified in two of the 100 analysed food fragments, and a tuber of *Bolboschoenus glaucus* (as identified by Wollstonecroft et al. 2008; 2011) which so far has been found to be a component of three food fragments.

The further identification of *Triticum* spp. has not been possible yet, however in accordance with the macrobotanical remains from Çatalhöyük, the most likely wheat ingredients must have been either *T. monococcum, T. dicoccum*, ‘new type’ glume wheat or *T. aestivum*. Whether there were differences in the use of these cereals for food preparation between houses at the site is currently unknown, but we have clear archaeobotanical evidence of the abundant presence of these species throughout the Çatalhöyük sequence, at least for the North and South areas. In general, among the wheats, *T. dicoccum*/new type grains and glume bases outnumber those of *T. monococcum* and *T. aestivum*, which suggests that *T. dicoccum*/new type glume wheat was predominant throughout the sequence. Additionally, in the South and North areas, hulled barley is absent from early levels; it appears in Level South Q and North/4040 G and continues very sporadically in later levels (Bogaard et al. 2013). However, in the TP area, preliminary assessment indicates that hulled barley is more frequent (Bogaard et al. 2013; Bogaard and Charles, personal communication). In relation to the possible range of pulses, *Pisum* is the most common, *Lens* and *V. ervilia* occur in 10–20% of samples, while *Cicer arietinum* (chickpea) occurs in a small minority of samples and in early levels at Çatalhöyük (Bogaard et al. 2013). The presence of *Descurainia sophia* throughout is constant, as large quantities of this oilseed were found in storage features in the South and North excavation areas (Fairbairn et al. 2007; Bogaard et al. 2013). Although the specific uses of this *D. sophia* at Çatalhöyük have not yet been explored, its intentional addition as an ingredient to the Çatalhöyük food would be among the earliest evidence of use of condiments in the Neolithic.

Although specific proportions of the cereals and pulses in the food fragments cannot be quantified due to the small surfaces observed in the analyses, there is a clear dominance of cereals and wheats in particular among them. Also, wheat particles outnumber barley components in samples from the North and South areas, while barley particles are more frequent among samples from the TP area. In relation to this, preliminary archaeobotanical results from burnt storage rooms in the TP area suggests a greater presence of barley (mostly naked) and ‘new type’ wheat than in the South and North areas. These patterns could be interpreted as possible changes in ‘culinary choice’, with an increase in the use of barley for food preparation towards the end of the Neolithic sequence at Çatalhöyük. However, the analysed contexts from TP are still very limited and further investigation is needed to assess the possible presence of other cereal types.

### Food preparation processes

Based on the typological datasets created for this study and observations of the archaeological materials and their comparison with the experimental specimens, some preliminary conclusions can be drawn about the possible food preparation methods at Çatalhöyük East. We were able to identify four types of food matrices among the food fragments recovered from the North, South and TP areas, of which three were observed to be analogous to the internal structures of experimentally prepared dough, bread and porridge. While we see a varying presence of dough and bread-like fragments corresponding with Matrix 1 and Matrix 3 from the earliest Neolithic level South G to South S/4040J, porridge-like fragments like Matrix 4 have only been identified among the samples recovered from the latest Neolithic levels of the site from level South Q–North H onwards. While there are similar frequencies of dough-type and bread-type fragments throughout the South and North sequences, most of the TP food fragments (29 of 31) had a porridge-like microstructure, with large visible fragments of plant particles such as grain fragments, big bran areas, etc., cracks and channel voids.

The measured fragment sizes of pulse and cereal tissues indicate that the main component used for the preparation of the foods present in Matrices 1, 2 and 3 must have been fine cereal and pulse flour which had been sieved after grinding, or it had at least been subjected to repeated grinding. No tissue parts were visible to the naked eye; only five of the measured components exceeded a maximum length of 500 µm, while the major part measured between 100 and 300 µm. According to Dickson (1990), without sieving, large bran fragments of 500 µm length and above should have been visible in the respective cereal product. On the other hand, the measured broken grains, bran and chaff particles in Matrix 4 foods indicate that coarse grain must have been the main ingredient. This pattern might be due to shorter grinding periods or else due to an intentional choice of coarse grain over fine flour for the preparation of these foods. The possibility that these materials were not sieved has been considered, however due to the absence of small particles (100–400 µm) they do not show a deliberate mixture of fine flour and coarse grain. Altogether, the fact that all visible particles range between 500–1,500 µm, in addition to the arrangement and type of voids and a lack of crumb structure, indicates a different cooking technique more related to a porridge or gruel preparation rather than bread baking (Dickson 1990; Lannoy et al. 2002).

## Conclusions

Cereal agriculture was one of the key developments of the Neolithic in the Near East, which is considered to have supported growing populations by means of the storability of cereals and their suitability for high calorie foods. Among the characteristic cereal-based foods are different types of porridge-like and bread-like products, the latter based on flour preparation and baking. Bread has been suggested to be a characteristic of the cultural traditions of the Near Eastern Neolithic, in contrast to cereal-based Neolithic food systems in some other world regions (Haaland 2007; Fuller and Rowlands 2011). The Near East is one of a handful of world regions in which crop domestication took place in communities without pottery cooking vessels, in contrast to the early development of ceramics amongst foraging societies, for example in the African Sahara or in eastern Asia (Kuzmin 2013; Jordan et al. 2016). While flour production through grinding has long been inferred as central in Near Eastern cultural traditions, based on the preponderance of quern stones from before and during the Neolithic (Wright 1994, 2014; Fuller and Rowlands 2011), what has been less certain is when the preparation of bread began, and the relative importance of breads versus porridge or other preparations.

Bread is certainly central to the long-term cultural traditions in the Near East, in contrast to regions like Nubia (Haaland 2007) or eastern Asia where cereals were boiled earlier on and for cultural reasons (Fuller and Rowlands 2011). It was hypothesised that bread preparation may well have preceded cereal domestication, and may even be a factor in the preference for cereals with gluten protein, mainly *Triticum* and *Hordeum*, amongst early cultivars (Lyons and D’Andrea 2003; Rowlands and Fuller 2009; Fuller and Rowlands 2011). The social importance of bread as a cultural foodstuff may have contributed to the importance of cereal consumption and may have been one of the factors that promoted cultivation and domestication (Maeda et al. 2016). Clay ovens certainly developed during the course of the Pre-Pottery Neolithic of the Near East, as for example at Tell Sabi Abayad II in Syria (Akkermans and Schwartz 2003; Akkermans et al. 2006), perhaps having developed from earlier pit ovens (Cauvin 2000; Fuller and Rowlands 2011). Nevertheless, finding out when bread became a periodic or routine food requires hard evidence.

The methods presented in the current paper provide a means to establishing both the presence and frequency of bread-like and porridge-like products as a food stuff. Ideally these methods should be combined with other approaches, such as organic analyses of food crusts from pottery, where this is possible. Large scale archaeobotanical flotation programmes can be expected to produce amorphous charred fragments from the remains of prepared foods, even from the period before pottery. Some of these charred “food lumps” can be categorised by microscopic analyses as probably deriving from bread-like preparations, porridge-like preparations, or others. While there are some categories of food remains that can be classified only descriptively, but for which we do not yet have an analogue food preparation with which to identify them (Matrix type 2), many others can be categorised as probably deriving from unbaked dough (Matrix type 1), bread (Matrix type 3) or porridge (Matrix type 4). As illustrated by our case study material from Neolithic Çatalhöyük, some food remains can be inferred to derive from bread and dough throughout the occupation, but these predominate throughout the earlier phases of the site occupation (ca. 7100–6400 bc). This fits with the idea that bread was already established and important during the Pre-Pottery Neolithic prior to and during the era when the site was founded. Of interest is that porridge type remains become prominent in the later phases at the site (ca. 6400–6000 bc), which correlates with periods in which pottery use increased (Last et al. 2005, p 102; Yalman et al. 2013) and in which domesticated cattle became important (Russell and Martin 2013), and with a probable increase in the use of dairy products (Copley et al. 2005; Evershed et al. 2008; Pitter et al. 2013). These data suggest that diversification of cereal-derived foodstuffs beyond bread to include more porridges took place together with diversification in ceramics in the early stages of the pottery Neolithic.
